# Functional MRI connectivity of children with autism and low verbal and cognitive performance

**DOI:** 10.1186/s13229-018-0248-y

**Published:** 2018-12-27

**Authors:** Terisa P. Gabrielsen, Jeff S. Anderson, Kevin G. Stephenson, Jonathan Beck, Jace B. King, Ryan Kellems, David N. Top, Nicholas C. C. Russell, Emily Anderberg, Rebecca A. Lundwall, Blake Hansen, Mikle South

**Affiliations:** 1 Department of Counseling, Psychology and Special Education, Brigham Young University McKay School of Education, Provo, USA; 2 Department of Radiology and Imaging Sciences, University of Utah School of Medicine, Salt Lake City, USA; 3 Department of Psychology, Brigham Young University, Provo, USA; 4 Interdepartmental Program in Neuroscience, University of Utah School of Medicine, Salt Lake City, USA; 5 Brigham Young University Neuroscience Center and MRI Research Facility, Provo, USA

**Keywords:** Autism spectrum disorder, Functional connectivity, Imaging methodology, Language, Intelligence

## Abstract

**Background:**

Functional neuroimaging research in autism spectrum disorder has reported patterns of decreased long-range, within-network, and interhemispheric connectivity. Research has also reported increased corticostriatal connectivity and between-network connectivity for default and attentional networks. Past studies have excluded individuals with autism and low verbal and cognitive performance (LVCP), so connectivity in individuals more significantly affected with autism has not yet been studied. This represents a critical gap in our understanding of brain function across the autism spectrum.

**Methods:**

Using behavioral support procedures adapted from Nordahl, et al. (J Neurodev Disord 8:20–20, 2016), we completed non-sedated structural and functional MRI scans of 56 children ages 7–17, including LVCP children (*n* = 17, mean IQ = 54), children with autism and higher performance (HVCP, *n* = 20, mean IQ = 106), and neurotypical children (NT, *n* = 19, mean IQ = 111). Preparation included detailed intake questionnaires, video modeling, behavioral and anxiety reduction techniques, active noise-canceling headphones, and in-scan presentation of the Inscapes movie paradigm from Vanderwal et al. (Neuroimage 122:222–32, 2015). A high temporal resolution multiband echoplanar fMRI protocol analyzed motion-free time series data, extracted from concatenated volumes to mitigate the influence of motion artifact. All participants had > 200 volumes of motion-free fMRI scanning. Analyses were corrected for multiple comparisons.

**Results:**

LVCP showed decreased within-network connectivity in default, salience, auditory, and frontoparietal networks (LVCP < HVCP) and decreased interhemispheric connectivity (LVCP < HVCP=NT). Between-network connectivity was higher for LVCP than NT between default and dorsal attention and frontoparietal networks. Lower IQ was associated with decreased connectivity within the default network and increased connectivity between default and dorsal attention networks.

**Conclusions:**

This study demonstrates that with moderate levels of support, including readily available techniques, information about brain similarities and differences in LVCP individuals can be further studied. This initial study suggested decreased network segmentation and integration in LVCP individuals. Further imaging studies of LVCP individuals with larger samples will add to understanding of origins and effects of autism on brain function and behavior.

**Electronic supplementary material:**

The online version of this article (10.1186/s13229-018-0248-y) contains supplementary material, which is available to authorized users.

## Background

Exploration of brain function has relevance for understanding origins, prognosis, and treatment of autism spectrum disorder (AS) [1]. Few neuroimaging connectivity studies have included individuals with AS and low language and cognitive performance (LVCP), and none have focused on this population. Functional MRI autism studies (without sedation) present logistical difficulties for LVCP individuals; attention, receptive language, holding still, and ability to control anxiety are especially challenging in unfamiliar, time-sensitive environments [2, 3]. Nonetheless, better understanding of brain connectivity in the LVCP population may provide valuable insights, including information to determine if brain differences are more pronounced as symptom severity increases, or if LVCP and higher verbal and cognitive performance and autism (HVCP) groups show different functional connectivity patterns. Neuroimaging of LVCP individuals is a long-awaited contribution to autism research, but many centers have not possessed the resources to develop methodology and technology to accommodate low language or cognitive levels, severe anxiety, restricted interests, and repetitive behaviors in a scanning environment [3–5].

New behavioral support methodology from Nordahl, et al. [6] achieved success in structural imaging of 9–13-year-old LVCP individuals without sedation. They utilized a Board Certified Behavior Analyst to facilitate individualized behavioral techniques, repeated mock scanner sessions, motion sensors for positive differential reinforcement during scanning, and repeated scans to obtain acceptable levels of head movement. They were able to achieve excellent results in 18 individuals with an average IQ level of 65 (range 41–108), including 10 children with very low language performance (standard scores ≤ 40 on the Verbal Ability index of the Differential Ability Scales, Second Edition, range 26.7–40). Cox et al. [7] focused on reducing head motion in children with autism in-scan procedure tasks over multiple trials (range 3–67), but concluded that behavioral techniques may be best suited to individuals with autism and higher language and cognitive performance.

### Functional connectivity in AS

Multiple researchers have shown altered functional connectivity (both overconnectivity and underconnectivity) in adolescents, adults, and some children with HVCP. A review of this research [8] reports most commonly decreased long-range functional connectivity during various cognitive tasks as well as no-task or “resting state” conditions. However, studies in younger children with autism have also shown brain hyperconnectivity, possibly suggesting developmental evolution of brain connectivity with age or development [9–11] No prior connectivity studies have included comparisons between LVCP, HVCP and neurotypical (NT) groups, although some suggest a continuum of functional connectivity associated with symptom severity exists within HVCP and NT samples [12].

Recent attention has focused on connectivity within default and salience brain networks as indicators of autism that can predict prognosis. The default network, including medial prefrontal, precuneus, inferior and medial temporal, and temporoparietal junction regions, is associated with internal narrative and stimuli [13] while the salience network involves anterior cingulate and frontoinsular regions and is associated with response to novel stimuli [14]. Several studies reported increased functional connectivity in autism in some neural subsystems [4, 11, 15–21], although some are in discrete neural subsystems such as inhibitory connections with corticostriatal connectivity. These findings may represent decreased inhibition in autism [15, 22–24] or may represent aggregate effects of large networks due to increased local connectivity [25] rather than long-range connectivity between discrete regions [11].

Some differences in connectivity abnormalities in autism across the literature may be explained by methodological variations across experimental conditions [26]. Nonetheless, multisite data sharing such as the National Database for Autism Research (NDAR) and Autism Brain Imaging Data Exchange (ABIDE) initiatives [27, 28] has shown convergent results from 1000+ participants, demonstrating predominantly decreased interhemispheric and within-network functional connectivity in adolescents and adults, particularly involving default mode and salience networks [27, 29, 30], increased corticostriatal connectivity and variable short-range connectivity on the scale of millimeters [27].

An attractive unifying hypothesis is that increased synchrony across different networks (as a result of impaired network *segregation*) and decreased connectivity within excitatory connections from distant regions within networks (representing impaired network *integration*) may arise from abnormal excitatory/inhibitory balance within cortical circuits [10]. This could result in altered network integration and segregation that impairs the brain’s ability to effectively integrate information from disparate brain regions within cohesive networks [31]. These symptoms may be seen behaviorally in impaired social abilities and increased restricted and repetitive behaviors [10, 32].

Individuals with LVCP have been systematically excluded from fMRI studies because of logistical limitations. No prior studies report how brain functional connectivity may be altered in LVCP, an important cohort where treatment decisions (including early intervention) are particularly impactful and could be informed by neurophysiological data. In particular, it is not well understood whether LVCP brain pathophysiology is similar to, but more severe than what is seen in HVCP, or whether brain processes are distinctly different.

A couple of recent studies have included samples with IQ score ranges that include some individuals in what we have considered the LVCP range [33, 34]. The focus of these studies has not been on the LVCP population results, however. For example, Reiter et al. [34] analyzed samples taken from existing data, including data from ABIDE sites. Within this sample, a lowerperformance group with autism included a mean Full Scale IQ score (77 ± 6) matched to a very high-performance group with autism (mean IQ 123 ± 8). Their results between these two autism groups showed significant underconnectivity within the default mode network and the visual ventral stream. Their high-performance group differed from typically developing controls in decreased anticorrelations among default mode, salience, and task-positive regions.

We hypothesized that behavioral and anxiety-reduction techniques may facilitate collection of functional MRI data in LVCP individuals to better inform the study of brain differences and similarities across the autism spectrum. We looked at a wide range of ages (ages 7-17) with LVCP, alongside HVCP and neurotypical (NT) children for comparison. Since resources such as a behavior analyst, mock scanner, and motion sensors may not be universally available, we also adapted the Nordahl et al. procedures to provide moderate levels of preparation and support that may be accessible to most centers. Our goal was to scan 80% of LVCP participants successfully, with a minimum of 200 motion-free volumes defining a usable scan.

Our second hypothesis about possible differences between LVCP, HVCP, and NT populations is guided somewhat by existing data collected from HVCP samples, but since so little is known about LVCP individuals, we used a discovery approach without restriction to specific brain regions.

## Methods and materials

We present an approach for evaluating brain function and connectivity in an LVCP sample of young participants (ages 7–17). Our study made use of detailed intake, video modeling, behavioral and anxiety reduction techniques, noise-canceling headphones, and a high temporal resolution scanning protocol.

### Participants

Participants responded to social media and email invitations to local autism groups. Families completed a phone intake to predict comfort level with scanning procedures (see Additional file 1: Table S1). Specifically, we invited potential participants who are typically able to tolerate dental visits and haircuts, although two participants with a history of needing sedation at the dentist also attempted scans. MRI safety checklists were completed in advance of arrival for both parents and participants. Ten families completed intake but did not attempt scanning, because parents predicted their children would not be able to hold still for more than a few minutes in the scanning environment.

We recruited a total of 62 children and adolescents (ages 7–17). Twenty of these were NT children with no history of psychological concern or diagnosis. The other 42 had been previously diagnosed with AS. We verified diagnosis based on observations from the Autism Diagnostic Observation Schedule, Second Edition (ADOS-2) administered by research-reliable psychologists.

#### Behavioral measures

Performance for language, cognitive, and social domains was measured using a battery consisting of an appropriate IQ test and ADOS-2 administration. IQ levels were estimated by the Wechsler Intelligence Scales for Children, Fifth Edition (WISC-V) [35], Wechsler Abbreviated Scale of Intelligence, Second Edition (WASI-II) [36], or Differential Ability Scales, Second Edition (DAS-II) [37] using extended norms as necessary. Overall composite scores and verbal scores were extracted for use in further analyses. Correlations between verbal and overall composite scores was high (*r* = .945, *p* < .001). Choice of which ADOS-2 Module (1, 2, 3, 4) to administer was made based on observed language production. Module 4 protocols were scored using the updated algorithm [38]. Calibrated severity (comparison) scores were calculated for all participants.

#### AS group assignment

Division into categorical groups was determined by composite IQ score. Individuals with an autism diagnosis and IQ ≤ 79 were assigned to the LVCP group while those with AS and IQ ≥ 80 were assigned to the HVCP group. The cutoff of about 80 is consistent with many other studies of functional connectivity in autism + “high” cognitive performance including [27, 33, 39].

#### Participant dropout

Four LVCP participants were unable to continue scanning procedures after lying down on the scanner table; three of these had no functional verbal language, and the fourth had some language but usually required sedation at the dentist. Two participants (one LVCP, one NT) completed the scan session but excessive head motion prevented further analysis. Finally, two LVCP participants had excessive head motion on their first try then returned on another day for successful scans (with no time required for acclimatization).

#### Final sample characteristics

The final sample thus included 56 participants across the three groups: LVCP *n =* 17, mean IQ = 54, IQ range 25–77; HVCP *n* = 20, mean IQ = 106, IQ range 85–130; and NT *n* = 19, mean IQ = 112, IQ range 85–134. Table 1 details demographic and psychological test data for the three groups. Participant age was equivalent across all groups (*F* = .442, *p* = .645*)*, and ADOS-2 Calibrated Severity Scores were equivalent across the two AS groups (*t* = .995, *p* = .326). LVCP had significantly lower IQ composite scores than other groups (*F* = 84.122, *p* < .001), but no significant difference was found between the HVCP and NT groups (*p* = .644). These relationships were likewise true for verbal IQ scores only.

**Table 1:** Participant demographics, showing means (± SD) and [range]

	LVCP (*n* = 17)^1^	HVCP (*n* = 20)	NT (*n* = 19)^2^	Comparison
Male:female	14:3	15:5	14:5	–
Age (years)	12.26 (± 3.34) [7–17]	12.64 (± 2.87) [7–17]	11.76 (± 2.61) [7–17]	LVCP = HVCP = NT
IQ composite score^3^ (standard score)	54.00 (± 17.50) [25–77]	106.85 (± 13.64) [85–130]	111.76 (± 13.05) [85–134]	LVCP < HVCP = NT
Verbal score^4,5^ (standard score)	47.5 (± 20.09) [25–78]	103.63 (± 11.73) [85–126]	114.84 (± 13.8) [94–140]	LVCP < HVCP = NT
ADOS-2 Modules administered	Module 1 *n* = 5 Module 2 *n* = 8 Module 3 *n* = 4	Module 3 *n* = 16 Module 4 *n* = 4		
ADOS-2 comparison score^6^	7.94 (± 1.52) [4–10]	7.35 (± 2.01) [4–10]	–	LVCP = HVCP

*LVCP* low verbal and cognitive performance with autism, *HVCP* high verbal and cognitive performance with autism, *NT* neurotypical. ^1^Original group of 22 less 4 with unsuccessful scans and 1 with excess motion. ^2^Original group of 20 less 1 with excess motion. ^3^ IQ composite is the Differential Ability Scales-II General Conceptual Ability or Wechsler Full-Scale IQ. ^4^Verbal scores reflect Vocabulary subtest or Verbal index scores. Two scores from LVCP group were not obtained (verbal level estimated by ADOS Module 1 for adolescent and another with overall composite score of 49). The verbal score was not obtained for one individual in the HVCP group, IQ composite score = 88). ^5^Verbal scores were highly correlated with overall IQ scores, *r* = .945, *p* < .001. ^6^Autism Diagnostic Observation Scale, Second Edition, Calibrated Severity Score (comparison score), which is from a 10-point scale, where 0 = minimal-to-no evidence of autism, 10 = high evidence of autism. For all comparisons, significant differences indicate *p* < .001 and no significant differences indicate *p* > .05

### Preparation and support procedures

Our procedures are adaptable to most research and clinical settings. We utilized video modeling, an evidence-based practice for increasing skills in individuals with AS across levels of verbal or cognitive ability. Details of video modeling and other preparation and support procedures are shown in Additional file 1: Table S2. Participants were instructed to watch the video and listen to recordings of scanner sounds at least 3–5 times at home, before coming to the MRI facility. All participants watched the video again in the waiting room prior to giving assent. See Additional file 1: Table S3 for average reported viewing/listening times for video and audio files.

#### Anxiety reduction techniques

Upon arrival, the participant was informally assessed for anxiety via brief parent interview and interview of participant using “one-up” language. These results were then incorporated into individualized anxiety reduction plans. Comfort items (e.g., a stuffed animal) determined to be MRI safe were allowed in the scan. Participants were dressed in their own MRI safe clothing and so were not asked to change into scrubs. However, many asked to change into the “special socks” (with non-skid soles) that were modeled in the video. Parents who passed MRI safety checks and a research assistant were allowed to accompany participants at all stages of scanning and to maintain physical contact throughout the scan (e.g., touching legs) if desired. Parents and researchers could communicate verbally with the participant either vocally (in the scan room) or via headphones (from the control room). Exposure techniques in the scan room included allowing participants to watch live modeling (e.g., a parent modeling all scan procedures) and helping researchers operate the movement of the scanning bed prior to getting on. Participants were slowly moved forward through each step of the procedure, with references to the video model they had seen previously, only moving on when the participant was comfortable. If the participant liked weighted blankets, sandbags were positioned in preferred locations (typically on legs). Favorite YouTube videos were shown during the structural scan to increase comfort and interest. Because research assistants were present in the scan room, they visually monitored all LVCP and HVCP participants, looking directly into the bore to monitor movement. No participants were observed to be sleeping. NT participants were generally not accompanied. All participants watched videos during all scans and responded to verbal check-ins between scans, minimizing risk of falling asleep.

#### Noise-canceling headphones

Participants used standard foam ear plugs rolled and put into their ears. They were then fitted with OptoActive™ active noise-canceling headphones (OptoAcoustics Ltd., Tel Aviv), which both actively and passively cancel EPI gradient noise in the fMRI environment. We placed a headphone over each ear and held the headphones in place using a headband and/or foam wedges placed between the head and the coil cage, along with flat foam inserts that could be folded to fill the space between the headphone and the cage. Additional support against head movement was provided by inflatable positioning pads as necessary (Pearltec MRI/CT Multipad Plus, MagMedix, MA, USA) placed between the participant’s forehead and the head coil cage. The OptoActive™ noise-canceling microphone was also placed near the participant’s mouth so they could freely speak to researchers in the control room. Noise levels were reduced from ~ 100 db in the environment without ear protection to ~ 60 db (about the level of a restaurant conversation) when using both passive and active noise cancelation. The noise reduction also beneficially allowed participants to hear the audio track for the Inscapes video [40] shown during functional scans.

### Image acquisition

We mitigated effects of participant head motion by employing a high temporal resolution scanning protocol involving multiband echoplanar fMRI imaging (Siemens Trio 3T MRI scanner, 32 channel Siemens Head Coil, TR = 800 ms, TE = 33 ms, multiband factor = 8, flip angle = 52°, 2 × 2 × 2-mm resolution, whole brain coverage 72 slices, 1240 volumes per subject divided into two 8.5-min sequences). Structural MRI imaging consisted of 3D T1-weighted magnetization prepared rapid gradient echo imaging (MPRAGE) acquisition (TR = 20 ms, TE = 4.92 ms, flip angle = 25°, 1 × 1 × 1-mm resolution). All MPRAGE images were manually inspected prior to analysis, and two subjects’ data were discarded due to unacceptable motion artifacts on structural imaging that precluded accurate coregistration with functional imaging data. Using a relatively high temporal resolution allowed better temporal control of motion-free data segments that could be used for analysis and using relatively long acquisition periods (2 × 8.5 min = 17 min total) also facilitated identification of motion-free segments.

Functional MRI imaging was performed during presentation of audio-visual stimuli with low cognitive load to help mitigate head motion and anxiety during scan sessions and reduce drowsiness that might affect functional connectivity results. Stimuli consisted of the Inscapes movie paradigm, which depicts abstract shapes and slowly moving artificial scenes without social references or any narrative story and set to a soothing audio track. Previous results have shown that reproducible functional connectivity results similar to the standard resting state connectivity may be obtained using the Inscapes paradigm. In addition, head motion and drowsiness are decreased using Inscapes.

A sequential process for preparing raw imaging data for analysis was followed prior to data analysis. The sequence is shown in Additional file 1: Table S4 and consisted of motion realignment, coregistration to MPRAGE image, segmentation of MPRAGE image, and normalization of MPRAGE image to MNI template in SPM 12 software (Wellcome Trust, London) for MATLAB (Natick, MA). Subsequently, voxelwise nuisance regression included 6 subject motion parameters, degraded white matter, degraded CSF, and soft tissues of the face time series with bandpass filtering from 0.001 to 0.1 Hz. Degraded white matter and CSF time series were obtained by retaining all voxels at least one voxel removed from the boundaries of subject-specific restriction masks for white matter and CSF components from the segmented MPRAGE image. Because the use of global signal regression remains controversial in functional connectivity analysis (i.e., [41, 42]) we performed analysis both with and without the use of global signal regression. An additional processing stream also included the global signal as a regressor, which was obtained from the mean BOLD signal from all in-brain voxels determined by the union of tpm.nii images for gray matter, white matter, and CSF distributed with the SPM 12 software package.

For functional connectivity analyses, time series were extracted from concatenated volumes for each subject after removing frames before and after a head motion of greater than 0.2 mm, from 333 regions of interest (ROIs) comprising a parcellation of cortical gray matter from a published network parcellation of the brain [41]. This parcellation, http://www.nil.wustl.edu/labs/petersen/Resources.html, includes 333 Gy matter regions covering the cortex and 286 nodes ascribed to a functional brain network (47 regions ascribed to “none” including regions in temporopolar and orbitofrontal cortex, detailed in Parcels.xlsx at the above resource). For functional network analysis, we used 8 networks combining the twelve Gordon et al. [43] networks as follows: auditory, salience (Gordon et al. cingulo-opercular and salience networks), frontoparietal (Gordon et al. frontoparietal and cinguloparietal networks), default (Gordon et al. default and retrosplenial temporal networks), dorsal attention, ventral attention, sensorimotor (Gordon et al. sensorimotor hand and sensorimotor mouth networks), and visual, as previously described. Two levels of analysis were performed, one at the 8-network level and one at the 333 ROI level, to balance multiple comparisons and granularity of spatial information. Functional connectivity was estimated as the Pearson correlation coefficient between mean time series from each ROI, with connectivity estimates Fisher-transformed prior to group analyses. Network-level time series were estimated as the mean of all ROI time series within each network, which were Fisher-transformed prior to group analyses. Left-right homotopic connectivity was estimated for each ROI by the pairwise functional connectivity between each region and the corresponding region in the opposite hemisphere using the centroid closest to the mirror image of the region’s centroid.

Statistical analyses were corrected for multiple comparisons using false discovery rate correction across all ROI or network pairs used for respective analyses. Significant results were evaluated for relationship to age, sex, and number of motion-free volumes using a general linear model with age and motion-free volumes as continuous variables and sex as a discrete variable.

## Results

### Head motion

**Fig. 1:**
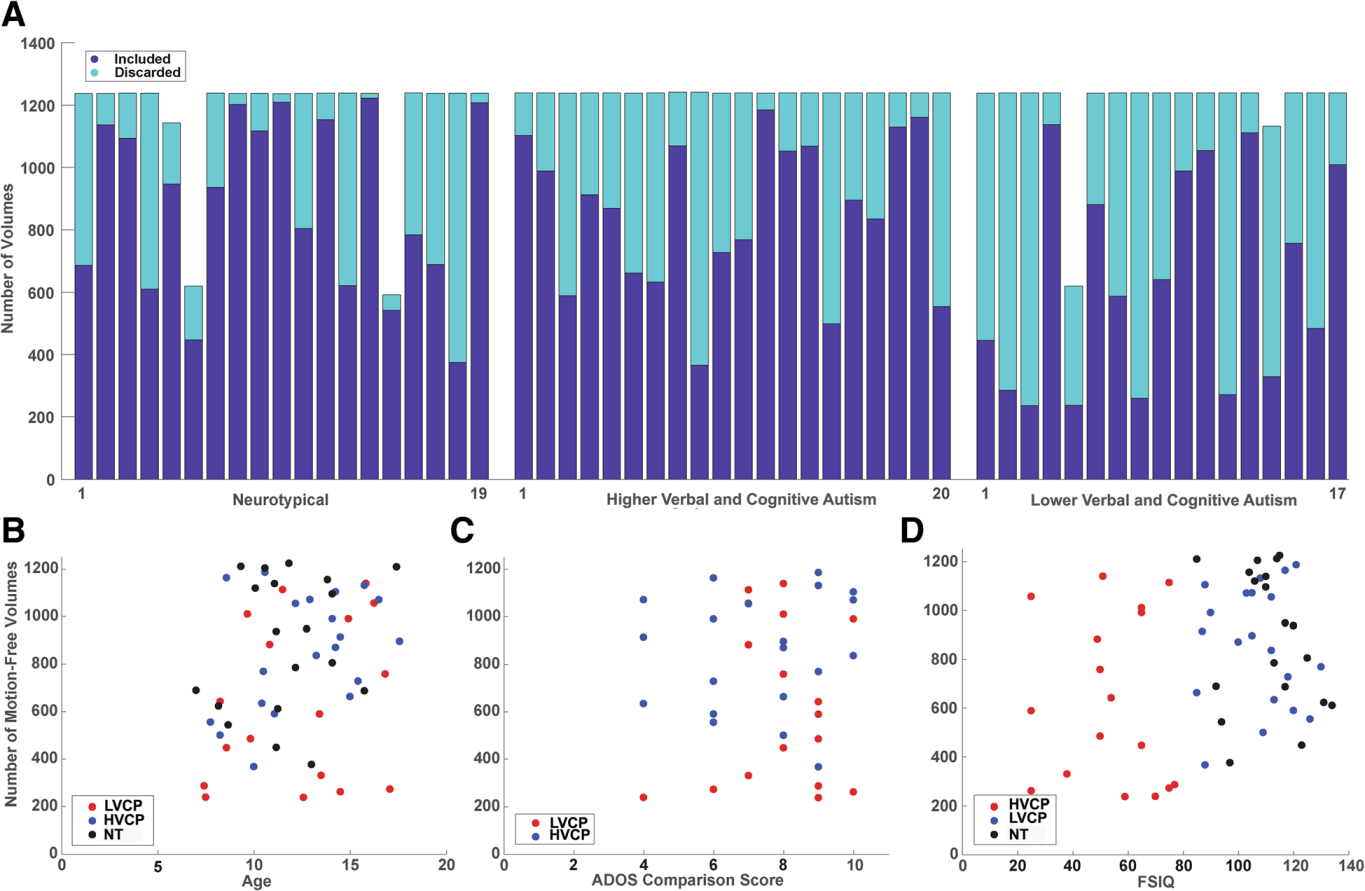
Subject head motion during fMRI scanning. **a** Bar graph shows for each subject the total number of volumes acquired and the number of motion-free volumes used in analysis. **b** Comparison of subject age and motion-free volumes for each sample. **c** Comparison of ADOS-calibrated severity (comparison) score and motion-free volumes for autism samples. **d** Comparison of IQ and motion-free volumes for each sample

As anticipated, participants in the LVCP group showed greater head motion during scanning than the HVCP or NT participants (3-group ANOVA: *p* = 0.024, mean number of motion-free volumes: LVCP 630 +/− 341; HVCP 854 +/− 244; NT 884 +/− 286). Mean framewise displacement prior to volume censoring differed significantly for the LVCP group (*p* = 0.00002, ANOVA) with mean framewise displacement: LVCP 0.31, HVCP 0.15, typical 0.13. These results are shown in Fig. 1, along with summary plots showing the relationship of head motion with age (*r* = 0.26, *p* = 0.047), ADOS-2 comparison severity score (*r* = 0.014, *p* = 0.93), and full-scale IQ (*r* = 0.24, *p* = 0.064). Two NT participants and one LVCP participant were able to tolerate only one 8.5-min BOLD sequence during scanning, and one NT and one LVCP participant had partial second scans. All participants were able to achieve at least 200 volumes of motion-free BOLD scanning. In particularly high motion subjects in our sample, head movements were interspersed relatively uniformly throughout the acquisition, with short epochs of motion-free frames. For example, in the most extreme case of a subject with 236 motion-free frames, the longest 2 epochs of unusable data were 37 and 28 s in duration with greater than 80% of unusable epochs less than 10-s duration.

In previous analyses, randomly censoring (scrubbing) greater than 50% of the volumes of a multiband acquisition sequence had negligible effects on reproducibility of functional connectivity results, although truncating the scan to shorter duration caused decreased reproducibility, indicating that the most important factor in single-subject reliability is the aggregate time in the scanner, allowing sampling of more brain microstates [44]. Since the underlying signals comprising functional connectivity measurements are slow, typically less than 0.1 Hz, interspersed measurements throughout the time in the scanner may allow meaningful sampling of these slow time-varying signals and estimation of functional connectivity measurements.

### Within-network functional connectivity

**Fig. 2:**
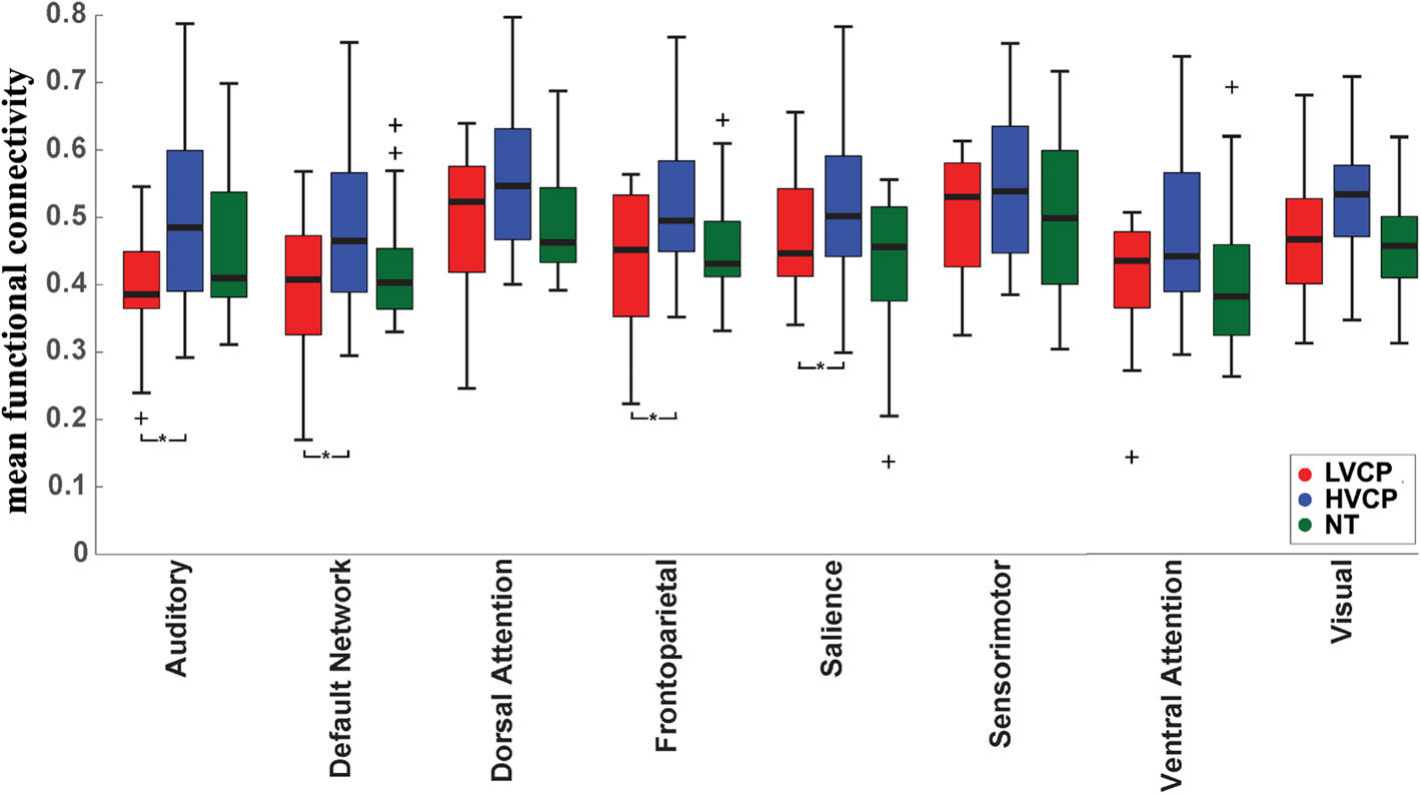
Within-network functional connectivity. Boxplots show mean functional connectivity across subjects for lower verbal, higher verbal, and neurotypical samples for sets of connections between ROIs within each of eight intrinsic connectivity networks. Asterisks demonstrate significant between-group differences (ANOVA)

To assess differences in functional connectivity within intrinsic connectivity networks, we examined the set of pairwise functional connectivity measurements between ROIs within each of the eight functional networks studied. For each participant, we calculated the mean functional connectivity between ROIs for each of these eight networks and used a three-group ANOVA to assess for group differences, with age, sex, and number of motion-free volumes as covariates. Significant between-group differences were observed for four of eight networks, shown in Fig. 2. Specifically, decreased within-network connectivity was observed (LVCP < HVCP) in participants for the default network (*F* = 3.45, *p* = 0.039), salience network (*F* = 3.93, *p* = 0.026), auditory network (*F* = 4.69, *p* = 0.014), and frontoparietal network (*F* = 3.83, *p* = 0.028). In this sample, neurotypical individuals showed intermediate within-network connectivity between LVCP and HVCP cohorts, without significant differences from either group.

### Between-network functional connectivity

**Fig. 3:**
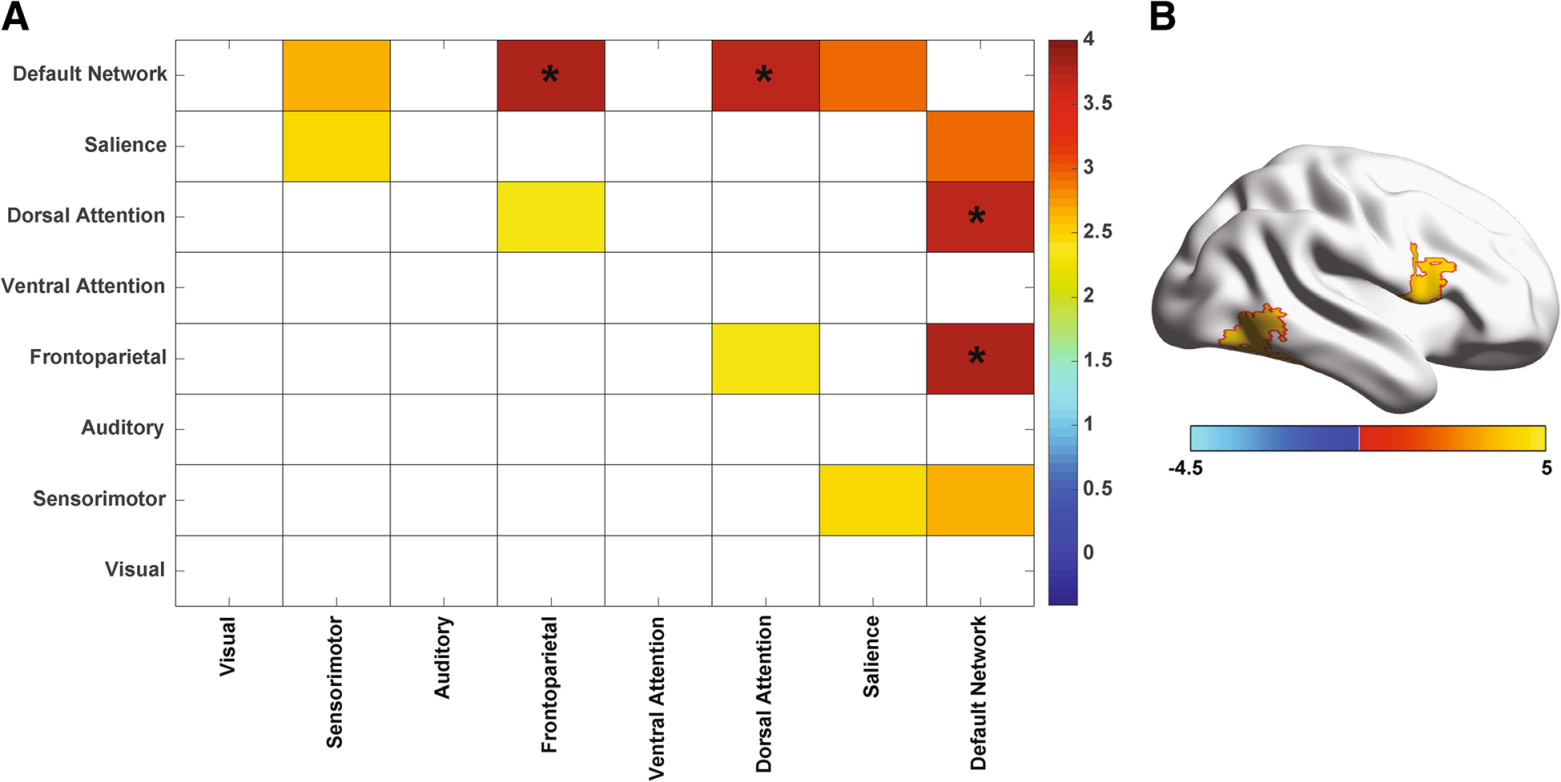
Between-network functional connectivity. **a** Pseudocolor plot shows networks for which the mean time series of each pair of networks showed significantly increased synchrony in minimally verbal autism subjects compared to neurotypical subjects, thresholded at *p* < 0.05, uncorrected. Asterisks indicate results satisfied false discovery rate *q* < 0.05 across all network pairs. **b** Significantly increased connectivity to the mean time series of the default network in minimally verbal autism participants relative to neurotypical participants. Colored regions satisfied false discovery rate *q* < 0.05 over all 333 ROIs compared to the default network

Mean time series for eight functional networks were obtained in each participant, and between-network functional connectivity (i.e., synchrony of the mean time series for each pair of networks) was evaluated for differences between LVCP and NT and between LVCP and HVCP participants. Results are shown in Fig. 3a. Connectivity was higher for LVCP participants than for NT participants between the default network and the dorsal attention network (two-tailed *t* test, *t* = 3.74, *p* = 0.0007), and between the default network and frontoparietal network (two-tailed *t* test, *t* = 3.67, *p* = 0.0008), with false discovery rate (FDR) *q* < 0.05 across all network pairs. These differences could not be explained by age, sex, or head motion in a general linear model including group, and these 3 covariates and showing persistent increased connectivity between default network and dorsal attention network (*F* = 5.40, *p* = 0.0075) and default network and frontoparietal network (*F* = 6.28, *p* = 0.0037). Significant differences in between-network connectivity were not observed between HVCP and LVCP cohorts.

Given the extensive literature characterizing functional connectivity abnormalities in the default network in autism, we specifically evaluated functional connectivity between this network and each of the 333 cortical ROIs analyzed, shown in Fig. 3b. Significantly higher functional connectivity was observed in LVCP compared to NT participants between the default network and three regions after false discovery rate multiple comparison correction. These regions were in the right temporo-occipital (two ROIs: MNI *x* = 47, *y* = − 52, *z* = − 12; *x* = 57, *y* = − 54, *z* = − 1) and right frontoopercular (MNI: *x* = 47, *y* = 8, *z* = 19) areas.

### Homotopic functional connectivity

**Fig. 4:**
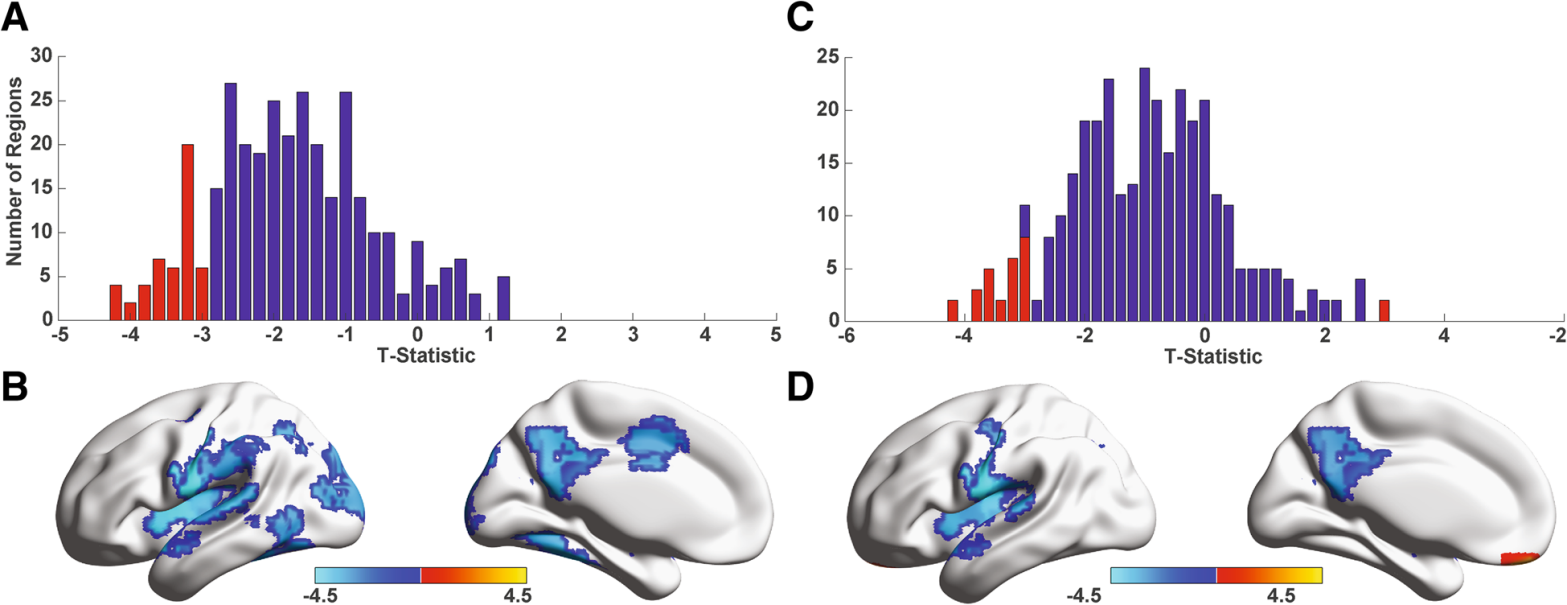
Decreased interhemispheric homotopic connectivity in minimally verbal autism sample. **a** Histogram shows *T*-statistic for all interhemispheric pairs of ROIs for lower verbal/cognitive (LVCP) and higher verbal/cognitive autism (HVCP) samples. Red bars show connections satisfying false discovery rate *q* < 0.05 across all region pairs. **b** Regions with significantly decreased homotopic connectivity for LVCP and HVCP samples is rendered on a template brain. **c** Histogram shows *T*-statistic for all interhemispheric pairs of ROIs for minimally verbal autism and neurotypical samples. Red bars show connections satisfying false discovery rate *q* < 0.05 across all region pairs. **d** Regions with significantly decreased homotopic connectivity for LVCP and NT samples are rendered on a template brain

Prior research on individuals with autism has also demonstrated decreased connectivity between left-right homotopic region pairs [27, 45], so we analyzed specifically whether similar connectivity differences were observed in our LVCP sample. Across the 333 ROIs compared to their interhemispheric homologs, we observed that 49 regions (FDR *q* < 0.05) exhibited decreased interhemispheric connectivity relative to HVCP participants, and 28 regions (FDR *q* < 0.05) exhibited decreased interhemispheric connectivity relative to NT participants, as rendered in Fig. 4. Widespread decreases in interhemispheric connectivity across the brain are also illustrated in histograms of *T*-statistics between LVCP and HVCP groups and between LVCP and NT groups. Significant differences in homotopic connectivity were not observed between HVCP and NT groups.

### GSR results

**Fig. 5:**
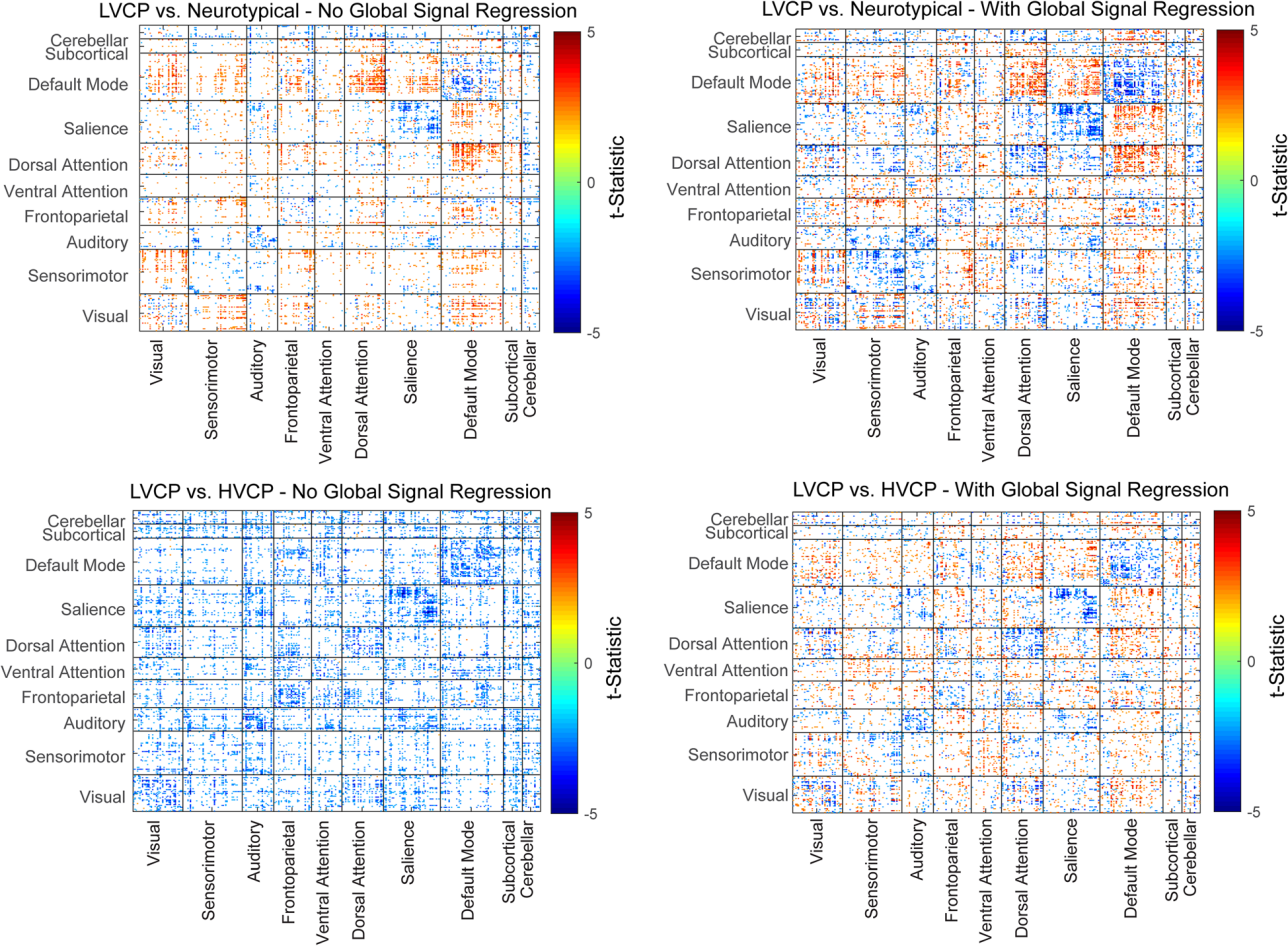
Group comparison of targeted networks using non-GSR vs. GSR analysis methods. To highlight effects of GSR across many connections, results were thresholded at *p* < 0.05, uncorrected, for display

Data from the GSR analyses show that there is a characteristic pattern of atypical connectivity in autism that is modulated by function, as shown in Fig. 5. In these analyses, individuals with autism show decreased within-network connectivity (especially homotopic and within the default and salience networks) but increased connectivity between the default and attentional networks (salience, dorsal attention, frontoparietal). Individuals with very low cognitive function when compared to those with high function show predominantly decreased connectivity across the brain (both within-network and between-network connections). When GSR is applied, which effectively normalizes the median connection to be zero, the effect of generalized decreased connectivity is removed, and the same pattern is seen for LVCP vs. HVCP as for LVCP vs. neurotypical individuals. In other words, if you adjust for the overall higher connectivity in HVCP individuals, you see the same pattern between LVCP and HVCP, suggesting that the “signature” of abnormal connectivity in autism is preserved and is worse in the LVCP group, implying this signature is more pronounced with greater disease severity.

### Effects of IQ on connectivity

**Fig. 6:**
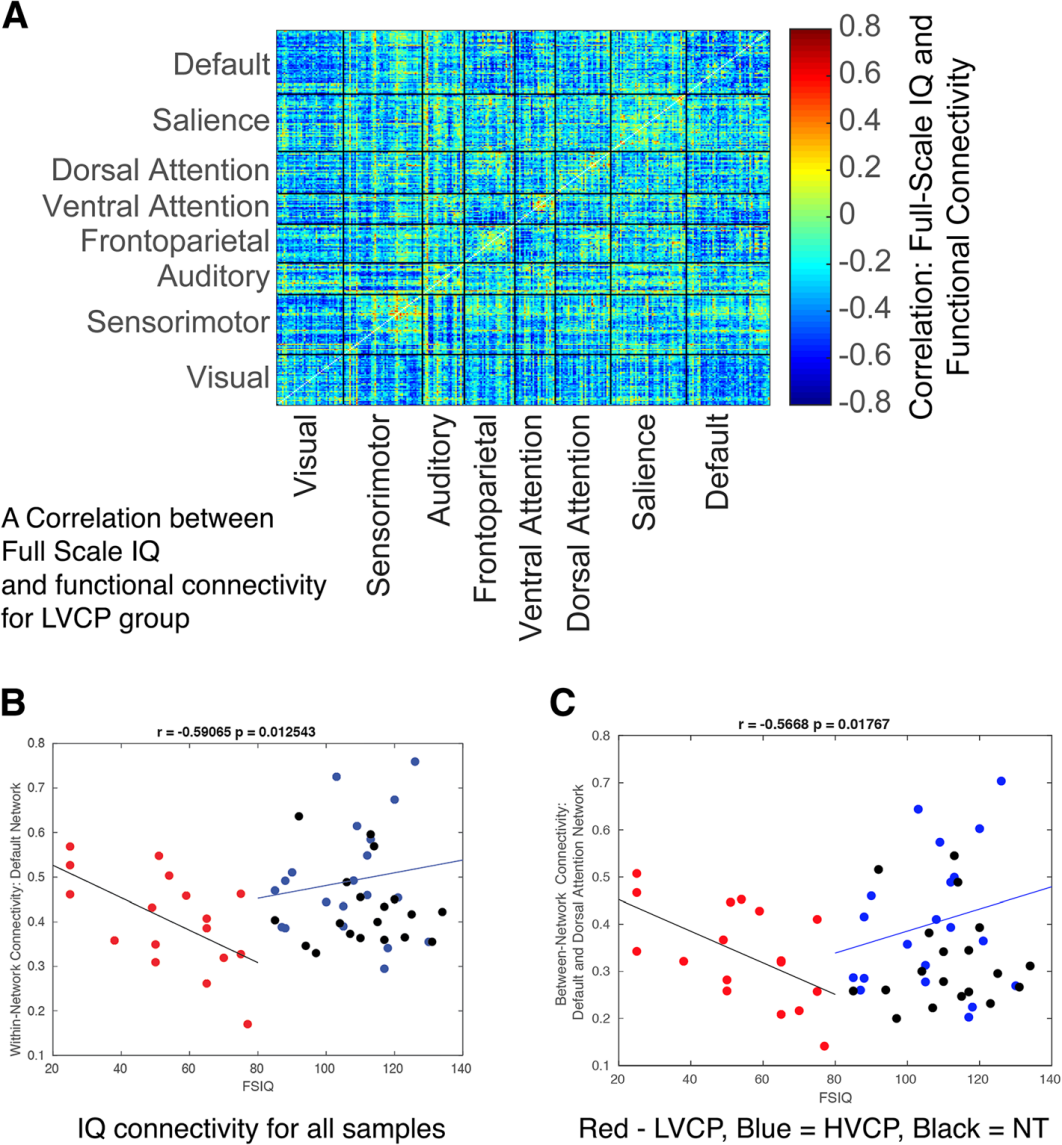
Dimensional effects of low IQ on functional connectivity **a** pseudocolor plot shows correlation between IQ and functional connectivity for each pair of corticocortical ROIs within the LVCP autism sample. **b** Mean within-network functional connectivity for the default network is compared to IQ for across three groups. **c** Between-network functional connectivity for the default and dorsal attention networks is compared to IQ across the three groups

For LVCP, we observed increased brain synchrony throughout corticocortical connections to be associated with lower full-scale IQ. Figure 6a shows Pearson correlation coefficients between full-scale IQ and functional connectivity between each pair of cortical ROIs for the LVCP group only. Almost all pairwise connections show higher functional connectivity for participants with lower IQ. These relationships could not be explained by head motion, as there was no relationship between number of motion-free volumes and full-scale IQ (*r* = − 0.061, *p* = 0.82) within the LVCP participants.

To explore these effects across the range of IQ performance in our sample, we examined between-group differences for two of the results with largest effect size by averaging functional connectivity for each subject for all pairs of ROIs in these two sets of connections: decreased within-network connectivity in the default network (Fig. 6b) and increased between-network connectivity between the default and dorsal attention networks (Fig. 6c),. Here, we observe the approximation of a U-shaped curve between IQ and connectivity: IQ is negatively correlated with connectivity for the LVCP group (especially true for the lowest IQ scores). However, these are positively correlated for HVCP and NT groups.

## Discussion

We have shown that methodological improvements, including using a combination of behavioral preparation, flexible supports, and improved scanning and analysis techniques, allow acquisition of functional MRI data from non-sedated children with LVCP, as young as age 7 and with IQ scores at the DAS-II basal of 25. This allows for more widespread study of a population with scarce functional neuroimaging data. Other than the noise-canceling headphones (a one-time purchase), techniques were not highly specialized and could be adopted by most research and health care settings. These methods were sufficient for individuals with minimal spontaneous or mostly echolalic language, although they did not extend as successfully to individuals with no functional verbal language or individuals with low ability to hold still for more than about a minute.

Multiband imaging sequences allowed for mitigation of head motion and physiological artifacts on the data, and analysis of concatenated motion-free segments allowed analysis of as much data as possible. Although the LVCP group had less motion-free data overall than other groups, of those who were able to fully enter the scanner, all but one provided adequate analyzable data (with a second try not available for that participant). This adds to the work of Nordahl, et al. [6] as evidence for possible scanning of individuals with autism from a more extensive range of language and cognitive performance, allowing a first theoretically sound, logical extension of fMRI analysis to a LVCP sample, compared to HVCP and NT data in the same study.

Generally, the LVCP children showed significantly reduced within-network connectivity relative to HVCP individuals, across auditory, default, frontoparietal, and salience networks. These results build on data with HVCP in multisite studies showing similar results for HVCP individuals compared to NT individuals [25, 29, 30]. Nevertheless, this effect has been inconsistent in the literature and our results do not reproduce these differences between HVCP and NT groups. Our initial findings may suggest that while reduced within-network connectivity, particularly in the default and salience networks, is more commonly observed than not in HVCP, this effect may become more pronounced when studying children and adolescents of lower functional ability.

Results with and without GSR complement each other to potentially inform one of the biggest controversies about functional connectivity in autism. While some similarities across previous studies are seen, some research groups have shown increased connectivity while others show predominantly decreased connectivity in autism. Our data show that there is a characteristic pattern of atypical connectivity in autism that is modulated by function. Individuals with autism show decreased within-network connectivity (especially homotopic and within the default and salience networks) but increased connectivity between the default and attentional networks (salience, dorsal attention, frontoparietal). As noted above, individuals with very low cognitive function, when compared to those with high function, show predominantly decreased connectivity across the brain (both within-network and between-network connections). When GSR is applied, which effectively normalizes the median connection to be zero, this effect of generalized decreased connectivity is removed and the same pattern is seen for LVCP vs. HVCP as for LVCP vs. neurotypical individuals. In other words, if one adjusts for the lower overall higher connectivity in HVCP individuals, one sees the same pattern between LVCP and HVCP, suggesting that the “signature” of abnormal connectivity in autism is preserved but is worse in the LVCP group, suggesting this signature is more pronounced with decreased cognitive and language performance level. We also note patterns in the similarity of how HVCP individuals most commonly differ from NT comparison groups in the literature, including overall decreased left-right homotopic connectivity [45], decreased *within*-network connectivity—especially for the default and salience networks—and variably increased connectivity *between* default and attention networks [46–48]. All of these findings are also associated with the LVCP group in our results.

Thus, our results provide one possible avenue for integrating competing reports of atypical connectivity in autism by suggesting that the relative pattern of connectivity abnormalities in autism may be uniform across the field, but since some groups have autism samples with relatively higher or lower function, their samples may pick up the overconnected parts and not underconnected ones (or vice versa). This is also dependent on technical factors as the results may be influenced by whether GSR was used. What ultimately differentiates individuals with autism who do well from those do not do well is whether they have decreased connectivity throughout the brain.

Dimensional analysis within groups shows that, for the LVCP group, there is a superimposed effect of low IQ resulting in higher global brain synchrony. Participants with the lowest IQ have higher connectivity across a broad range of corticocortical connections. This finding of general overconnectivity may represent overall poor differentiation and segmentation of brain regions, as previously seen in a sample of older adolescents and adults with Down syndrome with similarly low IQ [49]. It may be that lower verbal ability and IQ are associated with overall greater global synchrony and fewer task-specific activations. Electrophysiological neuroimaging (EEG) or magnetoencephalography (MEG) could evaluate this hypothesis by testing whether increased synchrony is related to hemodynamic aspects of the BOLD signal or synchronized neural activity.

Extensive variability exists across previous studies regarding whether there is increased or decreased connectivity in autism, and there is not consensus of which connections may be relatively under- or over-connected, and this is likely related to the heterogeneity of individuals with autism as well as interactions with age, IQ, and technical factors related to acquisition and preprocessing [26, 50]. The closest study to ours thus far is from Reiter et al. [34],which included higher and lower performance groups. Nonetheless their lower-performance group with autism included a mean Full Scale IQ score (77 ± 6) which was roughly equal to the highest IQ score in our LVCP group (IQ = 78) and more than 20 points (1.33 standard deviations) higher than the mean IQ of our LCVP group. Likewise the Reiter et al. high-performance group represented an extreme level of functioning (1^2/3^ standard deviations above the normative mean), thus direct comparison across our samples is problematic. We fully agree with their call for stratification of samples by general functional levels moving forward in functional connectivity autism research and anticipate that with analysis methods and supports outlined here and in Nordahl, et al. [6], more research centers will find increased access to imaging data in LVCP individuals.

In this report, there may be a competing effect of very low IQ. The participants with lowest IQ have relatively higher connectivity, despite lower connectivity for the LVCP compared to other groups. This suggests a generalized greater global synchrony in the lower IQ groups that may reflect a less specific response to environmental cues.

From a theoretical perspective, our results might be explained by decreased network segregation and integration [4, 10, 50–52]. Specifically, decreased within-network and homotopic connectivity are consistent with reduced integration of networks, and increased between-network connectivity of default and attentional networks is consistent with decreased segregation or differentiation of these networks.

This, in turn, might impair the ability to integrate information from disparate brain regions within cohesive networks [52] and contribute to some of the symptoms we see in autism. It has been proposed that such decreased network segmentation and integration may be related to imbalance in excitation and inhibition, thus shifting brain dynamics to local processing, with a detail-focused, “intense-world” physiology with weaker central coherence of cognitive processing [50, 53–57]. In this light, it is not surprising that individuals with LVCP show decreased interhemispheric and within-network functional connectivity compared to HVCP and NT individuals.

### Limitations

The ultimate goal of brain function research in autism is to discover predictors of individual performance to inform intervention. Nonetheless, considerable heterogeneity across the range of social, communicative, and intellectual functioning complicates the search for reliable individual difference factors. With our small sample, we are not able to determine predictors of individual performance with a high degree of confidence, and it is possible that the reason why LVCP vs. neurotypical between-network connectivity differences were observed while LVCP-HVCP differences were not is a combination of the small sample size, relatively higher effect size of connectivity differences in LVCP individuals, and greater heterogeneity of the HVCP sample.

Nevertheless, our study represents proof of concept that scanning LVCP children is possible and can add critical understanding of brain function in autism, although data loss from movement should be anticipated in LVCP groups. Studies involving the reproducibility of functional connectivity reassure that concatenated motion-free epochs of high temporal resolution fMRI data produce similar results to sustained datasets for an individual [44]. Nevertheless, effects of small head movements on functional connectivity data are complex, and our results should be interpreted in the context of data obtained from other methodologies such as sedated diffusion tensor imaging, optical, and electrophysiological techniques. The wide age range in our study could also be problematic in combination with the small sample size because connectivity likely changes across development [58]. A larger multisite study may improve sample size and allow for improved modeling of brain development associated with age.

Four individuals with extremely low functional language did not successfully scan. Scanning with individuals who had a history of difficulty with the dentist or haircuts was not generally attempted because of low ability to hold still and to avoid introducing aversion to the MRI environment (potentially affecting future health care) without the availability of more extensive supports. Thus, a degree of selection bias was present. More time in a mock scanner environment, with repeated attempts and behavioral support, have been shown to be effective [6, 7], but repeated trials for acclimatization and behavioral expertise may not be universally available. Some of our procedures, i.e., the multiband scan protocol and the active noise-canceling headphones, are also not universally available, although the scan sequences can be run on many machines and the headphones represent a one-time investment with large cost-benefit ratio.

## Conclusion

Many LVCP individuals can complete successful functional MRI scanning with moderate levels of support. Similar procedures may likewise be useful for other neuroimaging paradigms such as EEG and MEG. These procedures—including video modeling—are easily adaptable for other centers and populations. Our findings point to the influence of non-diagnostic factors, such as cognitive ability for understanding brain connectivity in autism and other neurodevelopmental disorders, while highlighting some similarities that may be consistent throughout autism regardless of level of verbal and cognitive ability. This project emphasizes the need for studying the entire spectrum of autism in order to elucidate individual difference factors related to etiology and outcome and to begin to develop more specific targets for intervention towards improved outcomes.

## Additional file

Additional file 1: **Table S1.** Intake screening questions. **Table S2.** Preparation and support procedures. **Table S3.** At-home preparation. **Table S4.** Image preprocessing. (DOCX 32 kb)

## Abbreviations

ABIDE: Autism Brain Imaging Data Exchange
ADOS-2: Autism Diagnostic Observation System, Second Edition
AS: Autism spectrum
BOLD: Blood oxygen level-dependent
CSF: Cerebrospinal fluid
DSM-5: Diagnostic and Statistical Manual of Mental Disorders, Fifth Edition
EEG: Electroencephalogram
EPI: Echoplanar imaging
FDR: False discovery rate
fMRI: Functional MRI
HVCP: Higher verbal and cognitive performance with autism
LVCP: Lower verbal and cognitive performance with autism
MEG: Magnetoencephalography
MNI: Montreal Neurological Institute
MPRAGE: Magnetization prepared rapid gradient echo imaging
MRI: Magnetic resonance imaging
NDAR: National Database for Autism Research
NT: Neurotypical
ROI: Region of interest
T: Tesla
TE: Echo time
TR: Repetition time
