# Supplementary material for: Functional MRI connectivity of children with autism and low verbal and cognitive performance
Source: Mol Autism. 2018 Dec 27;9:67. doi: 10.1186/s13229-018-0248-y (PMC6307191; doi:10.1186/s13229-018-0248-y)
Supplement: Supplementary file 1 — Table S1. Intake screening questions. Table S2. Preparation and support procedures. Table S3. At-home preparation. Table S4. Image preprocessing. (DOCX 32 kb) [file 13229_2018_248_MOESM1_ESM.docx]

Functional MRI Connectivity of

Children with Autism and Low Verbal and Cognitive Performance

***Tables***

**Table S1.** Intake screening questions.

|  | Typical Response Inclusion | Typical Response Exclusion |
| --- | --- | --- |
| Is there any metal (e.g., braces) in the child's body or your body? | No | Yes |
| Who diagnosed your child with autism spectrum disorder (autism, Asperger's, or PDD-NOS)? | MD or Psychologist | No Diagnosis |
| Does your child have any other diagnoses? | ADHD, OCD, anxiety, sensory processing disorder, intellectual disability | Known genetic disorders, seizure disorders, spina bifida |
| Is your child taking any medications? | ADHD, anxiolytic, antipsychotic medications or none | No exclusions^1^ |
| How much language does your child have? | Any verbal or sign language | No exclusions |
| How does your child do at the dentist? | Tolerates without sedation | Requires sedation^2^ |
| How does your child do with a haircut? | Tolerates reasonably well | Cannot tolerate |
| What does your child do when anxious? | Any response | No exclusions |
| What does your child do when afraid? | Any response | No exclusions |
| What kinds of sensory sensitivities does your child currently have? | Any response | No exclusions |
| When you tell your child to do something, which of the following is the most typical level of directions that he or she will follow immediately? 1-2-word command, phrase, or full sentence? | Any response | No exclusions |
| When you tell your child to do something, how much physical prompting do you usually have to give to help him/her follow the direction successfully? | Any response | No exclusions |
| When you think about all the directions you give your child throughout the day, what is your estimate of how often he or she follows the directions without delay? | Independent - range 0 - 80%; With assistance - range 20 - 100% | No exclusions |
| When your child is doing something, like watching a movie, how long can he or she continue watching (how long is his/her attention span for something he enjoys)? | < 20 minutes to 5 hours | No exclusions |
| When your child has to wait briefly for something (such as movie to start), how long can he or she typically be content to wait? | 2 - 45 minutes, most need explanation and reassurance | No exclusions |
| How long do you think your child would be able to lie still in an unfamiliar setting that includes some loud noises?  To help you make an estimate, consider that your child would have some practice hearing the noises and holding still in the setting. You would be allowed to be in the room and touch your child. Your child would be able to hear you talk and would also be able to hear others through a speaker. Your child would be able to look at a small mirror reflecting a video as he/she lay still. | 6 - 45 minutes | ≤ 5 minutes |

^1^Two participants in the HVCA group reported taking medications: risperidone and lisdexamfetamine dimesylate. ^2^Two participants requiring sedation at the dentist came to scanning visits. One scanned successfully, the other did not. Four others requiring sedation elected not to participate.

| **Table S2. Preparation and support procedures**  *See also:* [*Supplemental Video*](https://www.youtube.com/watch?v=jni4cE60dN0&feature=youtu.be) |
| --- |

| Procedure |  | Details |  |
| --- | --- | --- | --- |
| Video modeling |  | - 61 discrete steps, each explicitly modeled in the video. - model was an 11-year-old male with AS - researcher gives verbal explanations of scan procedures in person - placement of the head coil (the “Iron Man mask”) shots were from the perspective of what it would look like with the individual looking up as the top piece is connected - other segments showed the MRI machine and surrounding environment with full views of the model’s actions. - showed model entering the scanner bore - showed picture-in-picture image of the audiovisual stimuli the participant would see during functional scans (Inscapes) - actual scan time abbreviated (4:11) - requested to view at least 5 times prior to arrival |  |
| Audio exposure |  | - scanning functional and structural protocol sounds - requested to listen 5 times |  |
| Individual-ized anxiety reduction |  | - allowed to bring own MRI safe clothing and comfort item - live modeling of scan procedures by parent - participation in demonstration of raising/lowering scan bed allowed - physical contact with parent or research assistant allowed - slow pace of demands (1-hour scan appointments) to allow for acclimatization - favorite YouTube videos shown during structural scan - parents and researchers communicated verbally with participants vocally (in the scan room) or via headphones (from control room) |  |
| Positive reinforce-ment |  | - pairing of knee tap with “hold still,” and praise - immediate reinforcement for compliance with each step - delayed reinforcement (“treasure box,” cash, favorite activity) |  |
| Noise reduction |  | - foam ear plugs - OptoActive™ headphones (OptoAcoustics Ltd., Tel Aviv) - actively and passively cancel most echoplanar imaging gradient noise - reduction in surrounding noise from ~100db (no ear protection), to ~60db using both passive and active noise cancellation (about the level of a restaurant conversation). - headphones also allowed participants to hear audio included with videos shown during the scans. |  |
|  |  |  |  |
| Movement reduction |  | - foam padding inside head coil cage - inflatable positioning pads (Pearltec MRI/CT Multipad Plus, MagMedix, Massachusetts, USA) |  |
|  | | | |

| **Table S3. At-home preparation, number of times watching the video model and listening to scanner sounds** | | | |
| --- | --- | --- | --- |
| Mean (SD) [range] of occurrences | **LVCP (n=12)*** | **HVCP**  **(n=6)*** | **NT**  **(n=8)*** |
| Video | 4.4 (1.4) [2-6] | 3.2 (1.3) [1-5] | 1.6 (0.7) [1-3] |
| Audio | 3.6 (2.1) [0-8] | 3.6 (1.8) [2-6] | 1.7 (0.8) [1-3] |

*Although all participants were asked to view video and listen to audio, not all reported the number of times viewed/listened to. All participants with autism viewed the video in the waiting room before scanning (not included in these data).

| **Table S4. Image preprocessing** | | |
| --- | --- | --- |
| Process |  | Procedure |
| Blood oxygen level dependent effect (BOLD) image realignment |  | SPM12^1^: realign |
| Coregistration of BOLD and MPRAGE images |  | SPM12: coregister, estimate only, mean realigned image coregistered to MPRAGE image |
| Segmentation of MPRAGE image |  | SPM12: segment, thorough clean, 5 tissue compartments, modulated and normalized |
| Normalization of MPRAGE and BOLD images |  | SPM12: normalize, estimate and reslice, 2x2x2 mm resolution, Montreal Neuorological Institute (MNI) template |
| Nuisance regression |  | A general linear model (Matlab: glmfit.m) performed voxelwise regression of signal components:  --6 subject motion parameters,  --degraded white matter,  --degraded cerebrospinal fluid (CSF), and  --soft tissues of the face and calvarium as previously described (Anderson, Druzgal, *et al.*, 2011).  Simultaneous temporal filtering of voxelwise image time series and regressors performed with bandpass filter retaining components from 0.001 to 0.1 Hz. Global signal not included as a regressor, CSF and white matter regressors degraded by averaging from signal components at least one voxel removed from gray matter components (Saad *et al.*, 2012). |
| Scrubbing |  | Volumes before and after head motion of greater than 0.2 mm mean framewise displacement head motion were censored, with remaining volumes concatenated (Power *et al.*, 2012).^2^ |
| Post-hoc head motion comparisons |  | In addition to rigorous efforts to analyze motion-free data for each subject, the number of motion-free volumes was used as a subject-level regressor in connectivity analyses to quantify whether significant results obtained could be explained as a consequence of subject-specific head motion rather than between-group differences. |
